# Special Issue “Bacteriophage Genomics”: Editorial

**DOI:** 10.3390/microorganisms11030693

**Published:** 2023-03-08

**Authors:** Igor V. Babkin, Nina V. Tikunova

**Affiliations:** Laboratory of Molecular Microbiology, Institute of Chemical Biology and Fundamental Medicine, 630090 Novosibirsk, Russia

Virus genomics as a separate branch of biology has emerged relatively recently. Although the term “genomics” appeared only in 1986 [1], the history of genomics began in 1976, when the first ever complete RNA genome of bacteriophage MS2 (3569 nucleotides) had been sequenced [2]. During 1977, F. Sanger and coauthors first used their sequencing method to determine the first complete DNA genome of PhiX174 phage that infected *E. coli* [3]. Sequencing technologies have been further developed and automated, leading to the creation of hardware platforms for next generation sequencing (NGS) in the early 2000s [4]. So, the entire history of sequencing technology is closely related to phage genomes. PhiX174 is still one of the most commonly used positive controls for sequencing, including NGS.

The widespread use of NGS, both in metagenomic studies and in the sequencing of specific prokaryotic viruses revealed a striking diversity of bacteriophages. The development of sequencing techniques has led to the discovery of previously unknown bacteriophages of uncultivable prokaryotes. The size of newly discovered phage genomes varies from 3.5 kb in the case of microphages [5] to 735 kb in megaphages [6]. Bacteriophages have either DNA or RNA as their genome material. The striking diversity of bacteriophage genomes has led to the current predominance of the concept of their polyphyletic origin [7,8,9]. A large community of huge phages whose genome is more than 200 kb has recently been discovered [6,7,10]. These phages are found to be widespread in various ecosystems. In most cases, phages with extended genomes formed a separate clade according to phylogenetic analysis based on their specific genes [6]. Some of these genomes have a gene synteny that is different from most known phages; in some genomes, alternative genetic code is used. Some members of this phage community have even longer genomes than small bacteria [7,10].

The progress made in sequencing of the phage genomes has given a new look at their taxonomy. In 2008–2015, the *Autographiviridae*, *Myoviridae* and *Siphoviridae* families were revised based on their genomics [11]. Thereafter, the taxonomy of bacteriophages has been continuously modified and developed due to the appearance of new data on their genomes. Thus, while in 1999 the tailed phages had been divided into three families, which included 16 genera, and 30 species, in 2018 they were grouped into five families, 26 subfamilies, 363 genera, and 1320 species [7].Currently, phage taxonomy includes 47 families, 98 subfamilies, 1197 genera, and 3601 species (https://ictv.global/taxonomy; accessed on 20 February 2023). The process of phage taxonomy modification is not over yet.

The main result achieved in the bacteriophages genomics in recent years is the discovery of a significant mosaicism of their genomes. This indicates a widespread exchange of genetic information between phages by both homologous and non-homologous recombination [7,8,11]. This often makes the use of conventional methods of phylogenetic analysis unreliable, creating considerable difficulties in the taxonomy of bacteriophages and the study of their evolution. Network-based approaches, which are much more informative [7], should be used to solve such a problem.

Progress in expanding the bacteriophage genome databases has led to significant advances in both comparative and functional genomics of the viruses [7,8]. However, there are difficulties in the comparative genomics of phages. This is due to the lack of standard guidelines for the NGS phage genomes data submission; therefore, even closely related genomes are often submitted with different orientations and/or starting points. Such difficulties occur when linear genomes with terminal repeats are submitted into databases. To solve the above problems, various special softwares have been created for comparative analysis of bacteriophage genomes–ViPTree server [12], Gegenees [13], VIRIDIC [14], and other programs. These programs are based on matching phage ORFs and/or BLAST comparison. Another difficulty in studying phage genomes is the presence of modified nucleotide bases in some genomes [15].

In comparative genomics of temperate bacteriophages, a significant part of their genetic information may be extracted from databases of bacterial genomes. In this case, viral sequences are prophages integrated into the host genome [16]. When analyzing the prophage sequences, it should be taken into account that some of them belong to the cryptic prophages that never form active phage particles [17]. However, even such genomes are of interest for studying the evolution of viruses.

This Special Issue “Bacteriophage Genomics” focuses on the latest achievements and problems of bacteriophage genomics. To date, 14 articles have been published on comparative and functional genomics of these viruses [18,19,20,21,22,23,24,25,26,27,28,29,30,31]. Most articles published in the Special Issue deal with the functional and structural genomics of various phages, including the identification of phage endolysins, potentially useful for therapy. Phages and prophages of *Pseudomonas aeruginosa* and *Klebsiella pneumoniae* have been described in two articles [24,26]. These bacteria pose the greatest threat to human health, according to WHO (https://www.who.int/). In addition, viruses of pathogenic *Escherichia coli* strains have been analyzed in three studies [20,22,31] and in one article, phages of the *Campylobacter* genus are characterized [30]. *E. coli* and *Campylobacter* spp. are among the most common human and animal pathogens causing bacterial diarrhea. The published studies are in the trend of general interest in phage therapy of patients with infections caused by bacterial pathogens with multidrug resistance. It can be noted that five of the 14 publications in the Special Issue are devoted to prophages found in bacterial genomes, making an important contribution to the diversity of their hosts and to the exchange of genetic information between bacteria [19,25,26,28,30].

Importantly, some studies published in the Special Issue have proposed improved bioinformatic methods for annotation and analysis of phage genomes. Katelyn McNair and co-authors have developed a reliable method for in silico predicting protein-coding genes [18]. The developed workflow is important for comprehensive and accurate phage genome annotation. The method identifies both new genes and variants of existing genes. Certainly, this area of phage genomics requires further development since there are phages with extended genomes using alternative genetic code for stopping codons. This makes it difficult to correctly identify some genes when using traditional stop codons [6,10,32].

Moreover, prediction of host ranges of bacteriophages can be difficult in some cases. Currently, there is a growing interest in the therapeutic use of phage cocktails, which are combinations of phages able to lyse target bacteria. The use of such mixtures makes it possible to control infections for a wide range of pathogenic bacteria. Versoza and Pfeifer have performed computational prediction of the bacteriophage host range using modern bioinformatics tools [27]. Computer prediction of bacteriophage host ranges could significantly simplify the selection of the composition for such cocktails.

Although significant progress has been made in describing some viromes, such as those from the human intestine, the role of phages in many biotic communities remains poorly understood. In addition, it should be noted that the phage community is a dynamic system and genetic information is constantly exchanged both between phages and their hosts and between different phages, which is the main driving force of their evolution. The exponential increase in the number of phage genomes in databases leads to the need for constant revision of their relationships and taxonomy. This process will continue for the foreseeable future.

## Data Availability

Not applicable.
